# A Transport-Driven Conceptual Framework for the Biodegradation of FFF-Printed PLA/PHB Structures: Integrating Evidence from Complementary Experimental Studies

**DOI:** 10.3390/jfb17090438

**Published:** 2026-09-01

**Authors:** Alena Findrik Balogová, Marianna Trebuňová, Darina Bačenková, Radovan Hudák, Jozef Živčák

**Affiliations:** Department of Biomedical Engineering and Measurement, Faculty of Mechanical Engineering, Technical University of Kosice, Letná 1/9, 042 00 Košice, Slovakia; marianna.trebunova@tuke.sk (M.T.); darina.bacenkova@tuke.sk (D.B.); radovan.hudak@tuke.sk (R.H.); jozef.zivcak@tuke.sk (J.Ž.)

**Keywords:** PLA/PHB, biodegradation, fused filament fabrication, tissue engineering, transport processes, porosity

## Abstract

Biodegradation of fused filament fabrication (FFF)-printed poly(lactic acid)/polyhydroxybutyrate (PLA/PHB) structures is governed by complex interactions between material composition, structural design, and degradation environment. Although these factors have been extensively investigated, they are typically evaluated independently, limiting a comprehensive understanding of their combined influence on degradation behaviour. This study synthesizes experimental evidence from a series of complementary studies on FFF-printed PLA/PHB systems to develop a transport-driven conceptual framework for interpreting biodegradation mechanisms. The integrated findings show that structural porosity increases fluid uptake (approximately 10% in dense structures versus 20% in porous structures) and promotes greater mass loss (25–40% after 45 days), while material modifications, including plasticizers and ceramic additives, influence local pH evolution and mechanical stability during degradation. The degradation response was also strongly dependent on the surrounding medium, with PBS maintaining relatively stable pH, saline and Hank’s solutions promoting acidification, and urea-based media leading to alkalization. Collectively, these observations indicate that fluid absorption and diffusion consistently mediate the interactions between material composition, scaffold architecture, and degradation environment, thereby governing the progression of biodegradation. Based on these experimentally supported relationships, a transport-driven conceptual framework is proposed to provide a unified qualitative interpretation of biodegradation in FFF-printed PLA/PHB structures. Rather than presenting new experimental data, this work offers an integrative perspective that may facilitate the interpretation of degradation behaviour, support the rational design of biodegradable scaffolds, and provide a foundation for future quantitative and predictive models.

## 1. Introduction

Biodegradable polymer systems based on poly(lactic acid) (PLA) and polyhydroxybutyrate (PHB) are among the most widely investigated materials for tissue engineering because of their biocompatibility, tunable mechanical properties, and controlled degradation behaviour [1,2,3,4]. Combined with additive manufacturing technologies such as fused filament fabrication (FFF), these materials enable the fabrication of scaffolds with tailored external geometry and internal architecture, allowing structural and mechanical properties to be adapted to specific biomedical applications [2,5]. Since these scaffolds are intended to provide temporary mechanical support while gradually degrading and being replaced by newly formed tissue, understanding and controlling their biodegradation is essential for achieving predictable biological and functional performance. The degradation behaviour of biodegradable polymeric scaffolds directly influences not only their mechanical integrity but also the local physicochemical environment, thereby affecting cellular response and tissue regeneration. In addition, the degradation rate of biodegradable scaffolds must be carefully matched to the rate of tissue regeneration to ensure adequate mechanical support throughout the healing process. Consequently, understanding the factors governing biodegradation has become a major research focus in the development of functional biomaterials and patient-specific scaffolds [6,7].

Hydrolytic degradation of PLA/PHB systems is a complex process governed by multiple interacting variables. Previous studies have demonstrated that degradation behaviour is influenced by material composition, molecular structure, plasticizers, inorganic additives, scaffold architecture, porosity, and degradation environment [1,3,4,8,9,10,11,12,13,14,15,16,17]. These factors affect water penetration, hydrolytic chain scission, local pH evolution, mass loss, and changes in mechanical properties, ultimately determining the functional lifetime of biodegradable scaffolds. Considerable progress has therefore been made in understanding the individual contribution of these variables to polymer degradation.

Despite considerable progress in understanding polymer degradation, evaluating biodegradation remains challenging because different studies employ different degradation media, experimental conditions, and assessment criteria. In addition to mass loss and molecular weight reduction, current recommendations emphasize the need to evaluate fluid uptake, changes in mechanical properties, pH evolution, and degradation kinetics to obtain a comprehensive understanding of material behaviour. Recent systematic reviews have highlighted the importance of integrating these parameters rather than interpreting them independently [6,7,8,9,10,11,12,13,14,15,16,17,18,19].

The evaluation of biodegradable biomaterials is further supported by standardized testing procedures. ISO 10993-9 provides the general framework for the identification and evaluation of degradation products, while ISO 10993-13 specifies approaches for the identification and quantification of degradation products from polymeric medical devices [20,21]. In addition, ASTM F1983 provides guidance for assessing the biological response to absorbable biomaterials intended for implant applications [21]. Although these standards define testing principles and evaluation procedures, they do not establish a comprehensive framework explaining how material composition, scaffold architecture, and degradation environment collectively determine biodegradation behaviour [17].

Recent studies have demonstrated that transport-related phenomena, including fluid absorption, diffusion, and local chemical evolution, play a central role in controlling the degradation behaviour of biodegradable polymer systems. However, these processes are typically investigated separately within individual experimental studies, making it difficult to establish a unified interpretation of their combined influence [6,17,22]. To further investigate these interactions, our research group has investigated complementary aspects of biodegradation in FFF-printed PLA/PHB systems through a series of experimental studies addressing the effects of degradation environment, material modification, scaffold geometry, porosity, plasticizers, and ceramic additives [23,24,25,26,27,28]. Although each study focused on a specific research question, comparison of their findings revealed recurring degradation patterns that extended across different material formulations and experimental conditions. These observations suggested that the individual variables were not acting independently but were consistently interconnected through common transport-related phenomena governing fluid penetration, diffusion, and local chemical changes within the material.

This observation motivated the present work. Rather than presenting new experimental data, this study synthesizes experimental evidence obtained from these complementary investigations to develop a unified conceptual interpretation of biodegradation in FFF-printed PLA/PHB structures. The proposed framework emerged from the integration of experimental observations rather than from theoretical assumptions and is intended to explain how material composition, structural design, and degradation environment collectively influence degradation behaviour through transport-mediated processes.

The aim of this work is therefore to synthesize experimental findings from complementary studies and to propose a transport-driven conceptual framework that qualitatively explains the interactions between material composition, scaffold architecture, and degradation environment during biodegradation of FFF-printed PLA/PHB systems. By integrating previously independent observations into a unified interpretation, this framework provides a broader perspective on degradation mechanisms and may support the rational design of biodegradable scaffolds as well as future quantitative modelling of transport-controlled degradation processes.

Table 1 summarizes the complementary experimental studies that form the basis of the proposed transport-driven framework. Although each study addressed a specific aspect of biodegradation, together they reveal consistent relationships between material composition, structural design, degradation environment, and transport-mediated degradation behaviour. The synthesis of these observations provides the experimental foundation for the unified conceptual framework presented in this work.

## 2. Experimental Evidence Underpinning the Proposed Framework

The transport-driven conceptual framework proposed in this study is based on the synthesis of experimental evidence obtained from five complementary studies previously performed by the authors [23,24,26,27,28]. Similar approaches integrating complementary experimental observations have recently been recognized as valuable for improving the interpretation of biodegradation behaviour in biodegradable polymer systems [7,8]. These studies systematically investigated the effects of material composition, structural design, and degradation environment on the biodegradation of FFF-printed PLA/PHB systems.

The experimental scope of the individual studies is summarized in Table 1. Collectively, they examined the influence of plasticizers and ceramic additives, scaffold porosity and geometry, and different degradation media on fluid absorption, pH evolution, mass change, and mechanical behavior during degradation. Although each study addressed a specific research question, together they provide a comprehensive experimental basis for identifying common degradation patterns across different material systems and experimental conditions. These experimentally observed relationships are consistent with previous studies demonstrating that scaffold architecture, porosity, and degradation conditions collectively influence fluid transport, mechanical stability, and degradation kinetics of biodegradable scaffolds [29,30,31].

Comparison of these experimental findings revealed that the observed degradation behaviour could not be consistently explained by any single factor alone. This observation is consistent with previous studies demonstrating that structural voids, internal architecture, and diffusion processes jointly regulate the degradation behaviour of FFF-fabricated biodegradable polymers [29,32]. Instead, recurring relationships indicated that transport-related phenomena, particularly fluid absorption and diffusion, mediate the interactions between material composition, structural design, and degradation environment. These observations form the experimental basis of the transport-driven conceptual framework presented in this work.

Unlike a conventional review, this work does not aim to comprehensively summarize the existing literature but rather to integrate complementary experimental evidence within the context of current knowledge into a unified conceptual framework.

## 3. Key Experimental Observations

The synthesis of the experimental studies identified several recurring observations that consistently appeared across different material formulations, structural designs, and degradation environments. Although the individual studies addressed different research questions, together they revealed common relationships governing the biodegradation of FFF-printed PLA/PHB systems. The following sections summarize these key observations, focusing on the influence of structural design, material composition, degradation environment, and the transport processes that connect these factors. 

### 3.1. Structural Design Regulates Fluid Uptake and Degradation

Structural design is one of the key factors influencing the biodegradation behaviour of FFF-printed PLA/PHB structures. Among the structural parameters, porosity plays a particularly important role by determining the accessibility of the degradation medium to the material. Experimental observations from our previous studies demonstrated that porous structures consistently exhibited higher fluid uptake than dense samples, accompanied by greater mass changes and accelerated degradation [26,28].

The increased absorption observed in porous structures can be attributed to their larger specific surface area and the presence of interconnected pathways that facilitate fluid penetration into the scaffold. Enhanced fluid transport promotes hydrolytic degradation throughout the material, resulting in faster degradation compared with solid structures. This behaviour is consistent with previous studies demonstrating that printing-induced structural voids and internal architecture influence degradation primarily by facilitating fluid transport and reaction–diffusion processes within FFF-fabricated biodegradable devices [29]. A similar relationship between scaffold architecture, water uptake, and mechanical degradation has also been reported for 3D-printed PLA bone scaffolds [25,33]. Comparable effects of scaffold architecture on degradation behaviour have also been observed in FFF-fabricated PLA/hydroxyapatite scaffolds, where interconnected porous structures promoted fluid penetration while maintaining mechanical functionality during degradation [32]. In our previous studies, fluid uptake increased from approximately 10% in dense structures to around 20% in porous scaffolds, while mass loss after 45 days reached approximately 25–40%, depending on scaffold architecture and degradation conditions [26,28].

**Figure 1:**
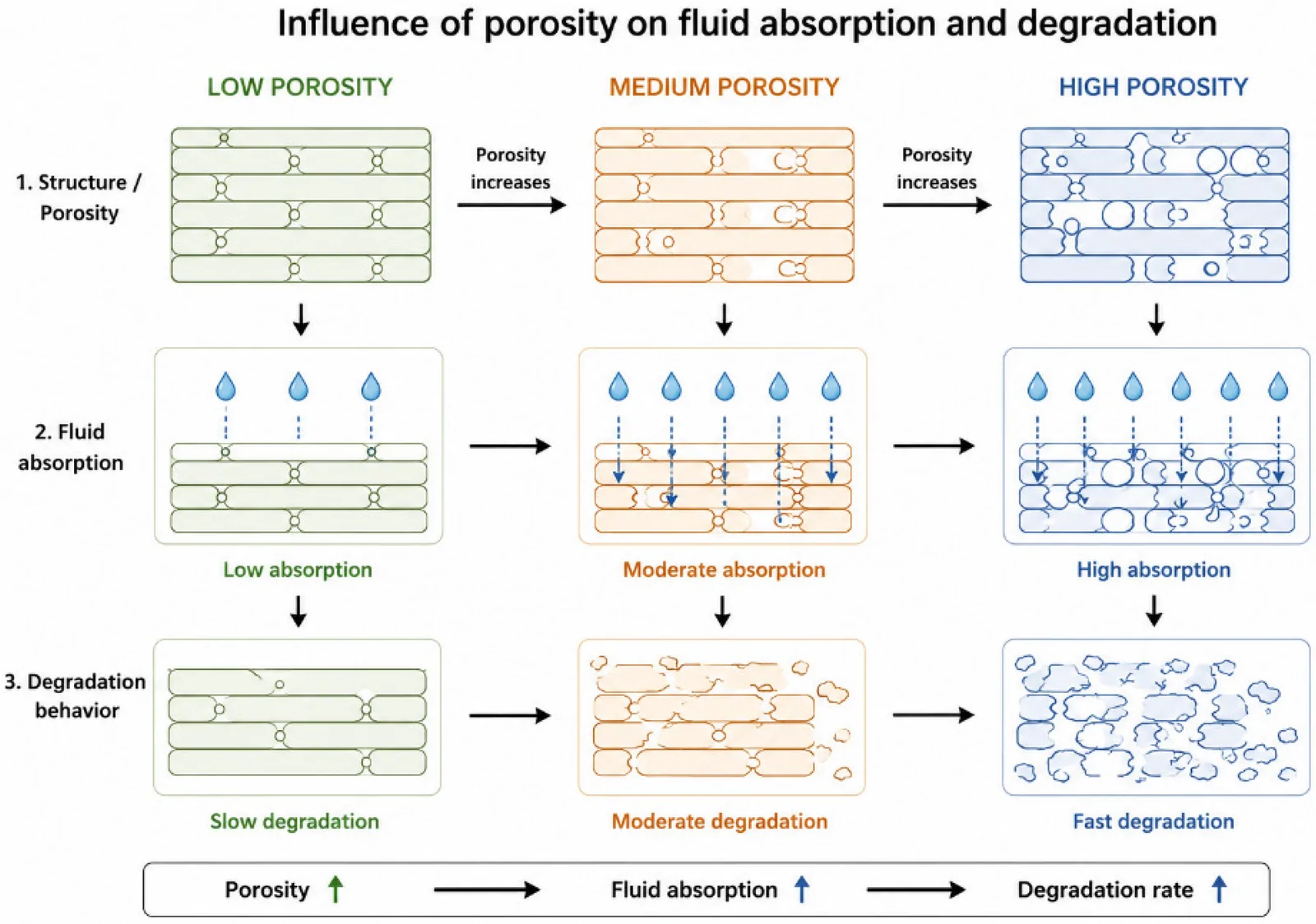
Conceptual illustration of the influence of structural porosity on fluid transport and biodegradation of FFF-printed PLA/PHB structures. Increased porosity enhances fluid uptake, facilitates transport processes, and promotes accelerated degradation. Arrows indicate the conceptual relationships between the individual processes.

The influence of structural porosity on fluid transport and subsequent degradation behaviour is conceptually illustrated in Figure 1. Increased porosity enhances the accessibility of the degradation medium, facilitates fluid absorption and diffusion, and consequently accelerates the biodegradation process. It should be noted that Figure 1 represents a conceptual illustration of the experimentally observed trends rather than a quantitative relationship.

In addition to porosity, the internal arrangement of deposited filaments may further influence degradation behaviour by modifying diffusion pathways and the accessibility of the degradation medium. Although this effect appears less pronounced than that of porosity, experimental observations suggest that filament architecture contributes to degradation primarily through its influence on transport processes [26,28]. Similar observations have recently been reported for FFF-printed biodegradable polyester blends, where printing parameters and internal architecture significantly affected water uptake, degradation behaviour, and mechanical durability during long-term environmental exposure [29].

These findings indicate that structural design should not be regarded solely as a geometric characteristic but also as a factor regulating fluid transport within the material. Consequently, scaffold architecture directly affects biodegradation behaviour by controlling the penetration and distribution of the degradation medium and should therefore be considered an active determinant of degradation kinetics rather than merely a geometric design parameter.

### 3.2. Material Composition Modifies Degradation Behaviour

Material composition represents another key factor influencing the biodegradation behaviour of FFF-printed PLA/PHB structures. In addition to the PLA/PHB matrix itself, modifications by plasticizers and ceramic additives alter fluid transport, local chemical conditions, and the evolution of mechanical properties during degradation [22,23,24]. Previous studies have demonstrated that plasticizers on hydrolytic degradation have been reported for PLA/PHB blends, where increased chain mobility promoted water diffusion and accelerated degradation kinetics [13].

Plasticizers such as oligomeric lactic acid (OLA), acetyl tributyl citrate (ATBC), and triacetin (TAC) are commonly incorporated to improve processability and flexibility. Experimental observations demonstrated that both the type and concentration of plasticizer significantly influenced pH evolution during degradation. While some plasticized systems exhibited pronounced acidification, higher OLA concentrations were associated with a more stable pH response, indicating that degradation behaviour depends on the interaction between material composition and the surrounding environment [23].

In addition to changes in local chemistry, plasticizers increase polymer chain mobility, thereby facilitating fluid uptake and accelerating hydrolytic degradation. These effects were also reflected in the biological response, where differences in cell adhesion and proliferation were observed depending on the plasticizer used [23].

Ceramic additives, particularly tricalcium phosphate (TCP), modified degradation behaviour differently from plasticizers. Similar effects of ceramic fillers on hydrolytic degradation and mechanical stability have also been reported for PLA-based composite scaffolds [33]. Rather than accelerating degradation, TCP contributed to a more stable mechanical response during degradation [24]. Comparable observations have been reported for PLA/hydroxyapatite scaffolds, where ceramic reinforcement improved structural stability while maintaining favourable degradation behaviour under physiological conditions [32]. Experimental observations showed that the compressive modulus of solid TCP-containing samples decreased by approximately 2%, 5%, and 11% after degradation in Hank’s solution, saline solution, and PBS, respectively. In porous TCP-containing samples, the corresponding reductions were approximately 6%, 8%, and 22%, demonstrating that both scaffold architecture and degradation environment influenced the mechanical stability of TCP-modified PLA/PHB structures [24].

These observations demonstrate that material composition affects biodegradation not only through intrinsic material properties but also by regulating transport processes, local chemical conditions, and the structural response of the material during degradation. The influence of material composition therefore extends beyond the chemical properties of the polymer itself and should be considered together with structural design and degradation environment when predicting biodegradation behaviour.

### 3.3. Degradation Environment Determines Chemical Evolution

The degradation environment represents another critical factor influencing the biodegradation of FFF-printed PLA/PHB structures. The composition of the surrounding medium affects fluid transport, local pH evolution, and the progression of hydrolytic degradation, resulting in different degradation pathways under otherwise comparable experimental conditions [26,27,28]. Comparable observations have been reported for biodegradable polymer systems exposed to different hydrolytic environments, where the degradation medium significantly influenced degradation kinetics, fluid uptake, and changes in material properties [11].

Experimental observations demonstrated distinct pH evolution trends depending on the degradation medium. PBS maintained a relatively stable pH throughout the degradation period, whereas saline solution and Hank’s solution exhibited rapid acidification to approximately pH 2. In contrast, urea-based medium showed a gradual increase in pH, reaching values close to 9. These differences were dependent on material composition and were already evident at the first measurement point, indicating that the influence of the degradation environment is established during the early stages of degradation [26,27,28].

**Figure 2:**
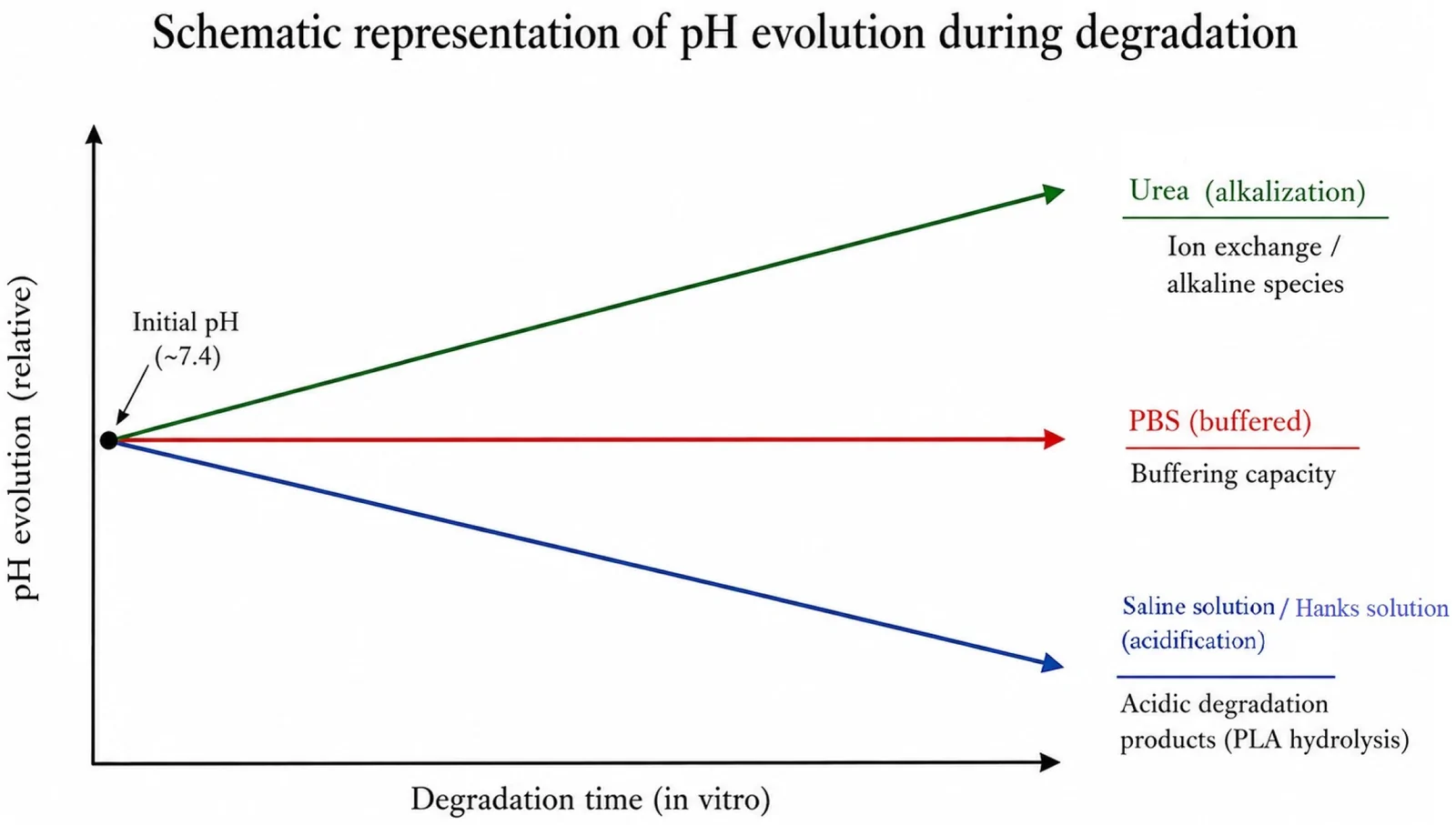
Summary of experimentally observed pH evolution trends during in vitro degradation of FFF-printed PLA/PHB structures in different degradation media. PBS maintained a relatively stable pH, whereas saline and Hank’s solutions promoted acidification and the urea-based medium resulted in alkalization. The figure summarizes experimental observations and is intended to illustrate the qualitative trends identified across the complementary studies.

The experimentally observed pH trends in different degradation media are summarized conceptually in Figure 2. It should be emphasized that Figure 2 illustrates the overall behaviour observed experimentally rather than representing a quantitative predictive model.

The distinct degradation responses observed in different media demonstrate that biodegradation cannot be interpreted solely as a material-dependent process. Instead, the surrounding environment actively influences local chemical conditions, thereby affecting degradation kinetics and the evolution of material properties [29]. These findings further support the need to consider degradation as the result of interactions between material composition, structural design, and environmental conditions. Consequently, identical scaffold designs may exhibit substantially different degradation behaviour when exposed to different physiological environments.

### 3.4. Transport Processes Link Material, Structure, and Environment

The experimental observations presented in the previous sections indicate that material composition, structural design, and degradation environment should not be considered as independent factors governing biodegradation. Instead, their effects are consistently connected through transport processes, particularly fluid absorption and diffusion, which regulate the interaction between the material and its surrounding environment [17,29].

Fluid absorption represents the initial stage of degradation, enabling water and ions to penetrate the material and initiate hydrolytic chain scission. This interpretation is consistent with previous studies showing that water absorption and diffusivity increase under conditions promoting hydrolytic degradation, thereby directly influencing the mechanical response and degradation kinetics of biodegradable polyesters [22,29]. The extent of absorption is determined by both scaffold architecture and material composition, while the degradation environment influences the chemical conditions under which these processes occur. Consequently, transport processes govern not only the rate of degradation but also the evolution of local pH, mass change, and mechanical properties [23,24,26,27,28]. Transport-controlled degradation mechanisms have also been reported for FFF-fabricated biodegradable devices, where printing-induced structural features governed fluid penetration and reaction–diffusion behaviour throughout the scaffold [29].

At the molecular level, hydrolytic degradation involves the cleavage of ester bonds, leading to a gradual reduction in molecular weight before macroscopic changes such as mass loss or mechanical deterioration become apparent [1,2,3]. In addition, restricted diffusion of degradation products may result in localized pH variations and autocatalytic degradation, particularly in less permeable structures where transport is limited [3,13]. These diffusion-limited phenomena have also been described using molecular modelling approaches, confirming the central role of transport in controlling degradation kinetics [15].

Taken together, these observations indicate that transport processes represent the common mechanism linking material composition, scaffold architecture, and degradation environment. This integrated interpretation provides the conceptual basis for the transport-driven framework proposed in the following section, in which transport processes are considered the central mechanism linking material composition, scaffold architecture, and degradation environment.

## 4. Transport-Driven Conceptual Framework

The synthesis of the experimental evidence presented in the previous sections led to the development of a transport-driven conceptual framework describing the biodegradation of FFF-printed PLA/PHB structures. Rather than considering material composition, structural design, and degradation environment as independent variables, the framework interprets biodegradation as the result of their continuous interaction mediated by transport processes.

**Figure 3:**
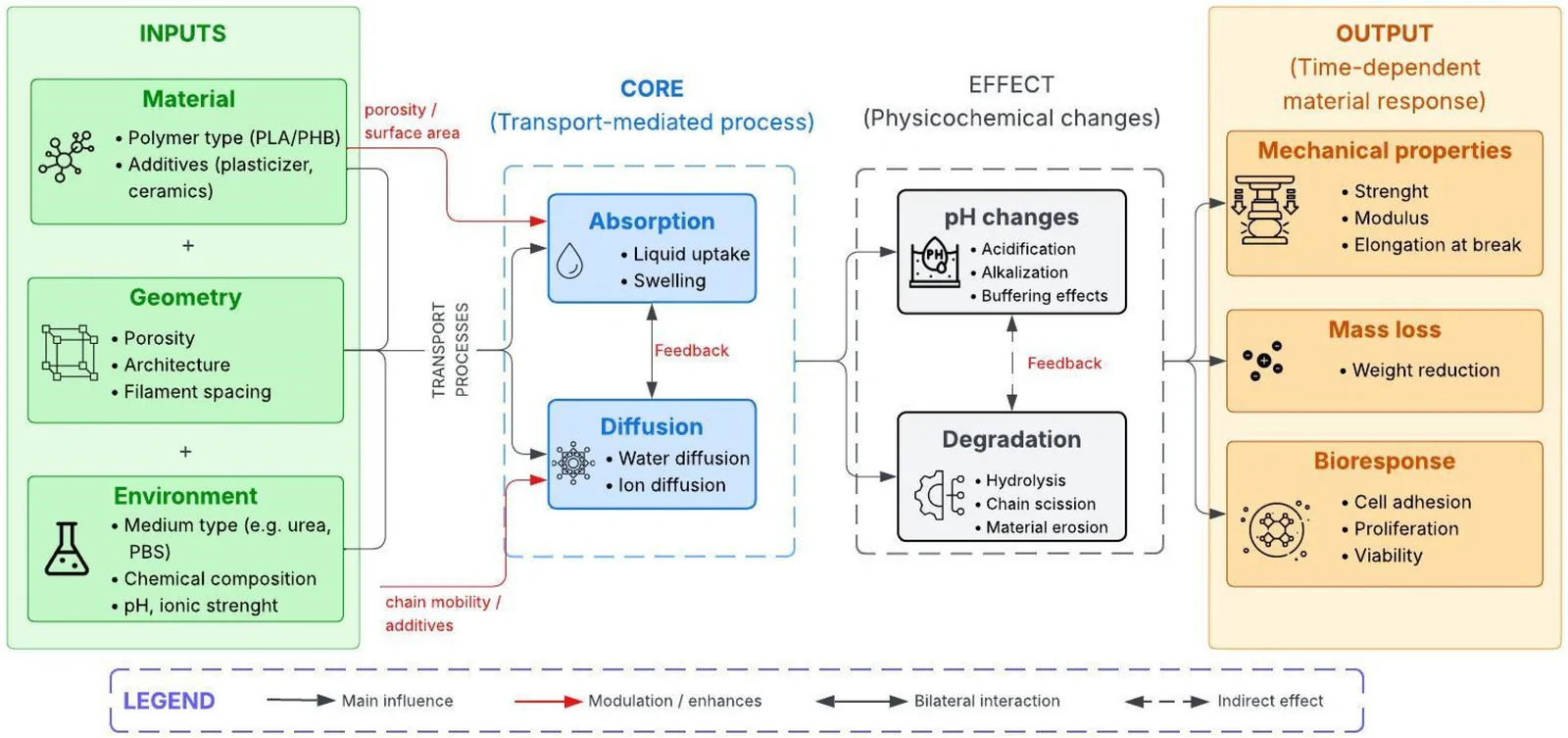
Transport-driven conceptual framework illustrating the interactions between material composition, structural design, degradation environment, and transport processes during biodegradation of FFF-printed PLA/PHB structures.

As illustrated in Figure 3, material composition, scaffold architecture, and degradation environment represent the three primary factors governing biodegradation. These factors collectively determine fluid transport into the material, including absorption and diffusion of water and ions, which initiates hydrolytic degradation and influences local chemical conditions. This interpretation is consistent with previous studies showing that transport pathways created by scaffold architecture and material composition govern degradation through reaction–diffusion mechanisms rather than through isolated material or structural effects [17,29].

Transport processes subsequently affect the evolution of material properties, including molecular degradation, mass change, mechanical behaviour, and pH development. Because transport is simultaneously influenced by material composition, structural design, and environmental conditions, it represents the central coupling mechanism connecting these factors throughout the degradation process.

It should be emphasized that the proposed framework is intended as a conceptual model integrating experimentally observed relationships rather than as a predictive mathematical model. Instead, it provides an integrative interpretation of experimentally observed relationships identified across the complementary studies summarized in Table 1. By integrating complementary experimental observations into a unified interpretation, the proposed framework provides a basis for future quantitative modelling and for the rational design of biodegradable scaffolds with controlled degradation behaviour.

## 5. Design Implications for Biodegradable Scaffolds

The proposed transport-driven framework highlights that the biodegradation of FFF-printed PLA/PHB scaffolds cannot be optimized by modifying a single parameter in isolation. Instead, predictable degradation behaviour requires the simultaneous consideration of material composition, structural design, and degradation environment, as these factors collectively regulate transport processes within the scaffold.

From a materials perspective, the selection of plasticizers and ceramic additives provides opportunities to tailor degradation behaviour by influencing fluid uptake, local chemical conditions, and mechanical stability. Similarly, scaffold architecture, particularly porosity and internal structure, can be adjusted to regulate the accessibility of the degradation medium and thereby control degradation kinetics. Comparable design strategies have been reported for FFF-fabricated PLA/hydroxyapatite scaffolds, where optimization of scaffold architecture and material composition enabled improved degradation behaviour while maintaining mechanical performance [32].

The findings also emphasize the importance of selecting appropriate in vitro degradation conditions when evaluating biodegradable materials. The distinct degradation responses observed in different media demonstrate that degradation behaviour cannot be generalized from a single experimental environment. Instead, degradation conditions should be selected according to the intended biomedical application and interpreted together with material composition and scaffold design. This recommendation is consistent with current standards for the biological evaluation of degradable medical devices, which emphasize that degradation testing should reflect the intended clinical application and relevant physiological conditions [19,20,21].

Overall, the proposed framework supports an integrated approach to scaffold design, in which material selection, structural architecture, and degradation environment are considered as interconnected parameters rather than independent design variables [4]. Such an approach may contribute to the development of biodegradable scaffolds with more predictable degradation behaviour and improved performance in tissue engineering applications. Such an approach may facilitate the rational design of biodegradable scaffolds with more predictable degradation behaviour, improved functional performance, and enhanced translational potential for tissue engineering applications [31].

## 6. Discussion

Biodegradation of FFF-printed PLA/PHB structures is governed by multiple interacting factors, including material composition, structural design, and degradation environment. While the influence of these individual variables has been extensively reported in the literature [1,2,3,4,5,6,7,8,9,10,11,12,13,14], the present work integrates complementary experimental evidence to provide a unified interpretation of their combined effects.

The synthesis of the five experimental studies consistently demonstrated that transport processes represent the common mechanism linking these factors. Structural porosity regulates the accessibility of the degradation medium, material composition modifies fluid uptake and local chemical conditions, and the surrounding environment determines the chemical pathways through which degradation progresses. Rather than acting independently, these variables collectively influence biodegradation through their effect on fluid absorption and diffusion.

The present framework extends previous experimental studies by integrating observations that have typically been investigated separately. Previous research has demonstrated the influence of material composition, scaffold architecture, or degradation environment individually, whereas the proposed framework emphasizes that these factors are functionally connected through transport processes. This interpretation does not replace existing degradation mechanisms but provides a higher-level conceptual explanation of how these mechanisms interact throughout biodegradation.

Unlike previous studies that primarily investigated the influence of individual variables on biodegradation, the present work integrates complementary experimental evidence into a unified conceptual interpretation. This interpretation is consistent with recent studies demonstrating the importance of diffusion-controlled degradation and reaction–diffusion phenomena in biodegradable polymer systems [4,17,29]. In addition, previous studies have independently demonstrated that scaffold architecture, material composition, and degradation environment each significantly influence biodegradation behaviour [6,7,8,13,14,15,16,17,18,19,34]. The present framework builds upon these observations by integrating these individual factors into a single conceptual interpretation based on transport-mediated interactions. The proposed framework does not introduce a new degradation mechanism but highlights transport processes as the common link through which material composition, scaffold architecture, and degradation environment collectively influence biodegradation behaviour.

The proposed framework is intentionally qualitative and should therefore be regarded as a conceptual basis for future quantitative modelling rather than as a predictive mathematical model. Furthermore, it is based on in vitro hydrolytic degradation of PLA/PHB systems fabricated by FFF and therefore should not be directly extrapolated to in vivo conditions or other biodegradable polymer systems. Future studies should combine experimental observations with transport modelling and degradation kinetics to develop predictive models capable of supporting the rational design of biodegradable scaffolds. Such quantitative models could facilitate the optimization of scaffold design for specific biomedical applications, reduce the need for extensive empirical testing, and improve the prediction of degradation behaviour under clinically relevant conditions. This perspective is consistent with recent reviews highlighting the need for standardized degradation assessment and the integration of transport mechanisms, material design, and degradation kinetics to improve the predictive design of biodegradable polymer systems [35].

Overall, the proposed framework contributes to the current understanding of biodegradable polymer systems by integrating complementary experimental evidence into a unified conceptual interpretation. Beyond explaining the interactions between material composition, scaffold architecture, and degradation environment, the framework may support future quantitative modelling, experimental design, and the rational development of biodegradable scaffolds with predictable degradation behaviour.

## 7. Conclusions

Biodegradation of FFF-printed PLA/PHB structures is governed by the combined effects of material composition, structural design, and degradation environment rather than by any single factor alone. By synthesizing experimental evidence from five complementary studies, this work provides an integrated interpretation of biodegradation and identifies transport processes, particularly fluid absorption and diffusion, as the central mechanism linking these factors.

The synthesized experimental findings demonstrate that structural porosity enhances fluid uptake and degradation, material modifications influence local chemical conditions and mechanical stability, and degradation media determine distinct degradation pathways. Collectively, these observations support the proposed transport-driven conceptual framework, demonstrating that transport processes constitute the common mechanism linking material composition, scaffold architecture, and degradation environment.

From a practical perspective, the proposed framework emphasizes the importance of simultaneously considering material composition, scaffold architecture, and degradation environment during the design and evaluation of biodegradable scaffolds. Although the proposed framework is qualitative and not intended as a predictive mathematical model, it provides an integrative basis for interpreting biodegradation experiments, supports the rational design of biodegradable scaffolds, and establishes a foundation for future transport-based quantitative models.

## Figures and Tables

**Table 1:** Overview of the experimental studies forming the basis of the proposed biodegradation framework, including material composition, structural design, degradation conditions, and key findings.

Study	Material Composition	Geometry	Environment	Duration	Key Findings	Study
**1**	PLA/PHB + plasticizer (~25%)	A/B, 100–50% infill	FR, urea	45 days	Strong pH divergence (acidic vs. alkaline), mass increase due to absorption	[27]
**2**	PLA/PHB/TPS + OLA/ATBC	50% infill	in vitro cells	14 days	OLA improved cell adhesion and viability, ATBC reduced bioresponse	[23]
**3**	PLA/PHB (85/15) + TAC (0–10%)	solid vs. porous	FR, PBS, Hank	195 days	Plasticizer affected pH and mechanical properties, PBS most stable	[24]
**4**	PLA/PHB + HA + TCP	solid vs. porous	FR, PBS, Hank	170 days	Ceramics stabilized mechanical properties, minimal pH variation in PBS	[25]
**5**	PLA/PHB (70/30) + OLA	solid vs. porous	FR, PBS, Hank	120 days	Porosity increased absorption and degradation, strong pH drop in FR	[28]

## Data Availability

The original contributions presented in the study are included in the article, further inquiries can be directed to the corresponding author.
